# Structural, Physicochemical, and Functional Properties of Waxy and Non-Waxy Foxtail Millet Starches

**DOI:** 10.3390/foods14173034

**Published:** 2025-08-29

**Authors:** Yuting Fan, Lei Chang, Yang Yao, Qin Dan, Pingping Zhang, Xinyi Li, Xiuzhu Yu, Shuangkui Du

**Affiliations:** 1 College of Food Science and Engineering, Northwest A&F University, 22 Xinong Road, Yangling, Xianyang 712100, China; yutingf@nwafu.edu.cn (Y.F.); changcl775@163.com (L.C.); 17389282268@163.com (Y.Y.); ddddyyq@163.com (Q.D.); 15229557391@163.com (P.Z.); xinyili1015@163.com (X.L.); xiuzhuyu@nwafu.edu.cn (X.Y.); 2 Shaanxi Union Research Center of University and Enterprise for Grain Processing Technologies, Yangling, Xianyang 712100, China

**Keywords:** foxtail millet starch, structural properties, physicochemical properties, functional properties

## Abstract

This study investigated the structural, physicochemical, and functional characteristics of foxtail millet starches (FMSs), including five non-waxy varieties (N-HXMS, N-LXMS, N-QZHS, N-JG21S, N-BLGS) and one waxy control (W-HJGS). All FMSs exhibited polygonal granules with surface pores and an orthorhombic crystalline structure (A-type X-ray diffraction pattern). Compared with the waxy FMSs, non-waxy starches exhibited higher amylose content (32.4–34.04%), reduced crystallinity (37.01–39.21%) and short-range molecular order, and lower hydration capacity and molecular weight (1.01 × 10^5^–2.81 × 10^5^ g/mol). The non-waxy FMSs also demonstrated enhanced resistance to mechanical shear, better structural stability, stronger recovery behavior, and reduced enzymatic susceptibility. Varieties like N-LXMS, with higher amylose and resistant starch contents (31.17%), are more suitable for functional foods targeting glycemic control, while W-HJGS, with higher swelling power (22.76 g/g) and solubility (92.30%), is well suited as a thickener. This study provides a foundation for future research on the modification of FMSs and their utilization as starch-based matrices in various applications, such as functional food development, biodegradable packaging materials, and targeted delivery systems for bioactive compounds.

## 1. Introduction

Foxtail millet (*Setaria italica* L.), one of the oldest cultivated grains, has gained renewed attention in recent years due to its superior nutritional components (rich in starch (≈63.2%), protein (9.28~15.8%), crude fiber (≈2.8%), and various minerals (≈2.7%)), resilience to harsh growing conditions, and potential contributions to food security [1]. Starch is the principal component of foxtail millet, playing a key role in its processing characteristics and end-use applications. Previous studies have demonstrated that foxtail millet starch (FMS), due to its antioxidant compounds, distinct crystalline structures, and good gelatinization properties, can serve as a functional food matrix, thickening agent, emulsifier, swelling agent in the food industry, and bio-based packaging such as bio-film production in industrial applications [2]. These unique characteristics (gelatinization and binding capabilities) make it a promising substitute for conventional starches such as maize, wheat, or potato starch (Table S1) [2]. It is well established that starches from different botanical origins exhibit distinct structural and physicochemical properties; for instance, corn starch typically shows A-type crystallinity with moderate viscosity, high gelation strength and a porous structure, while potato starch with a relatively higher pasting temperature exhibits B-type crystallinity [3]. However, FMS remains underutilized compared with starches from more commonly studied sources such as corn, potato, and rice [4].

A growing body of literature has compared starches of different botanical origins (e.g., corn, potato, barley, quinoa), consistently showing that their amylose content, crystal structure, and granule architecture have a potential relationship with distinct gelatinization and retrogradation behaviors [5,6,7]. Zhang et al. found that the amylose and amylopectin components of FMS influence its thermal properties and quality when used for porridge applications [5,8]. Some studies have attempted to improve the structural and physicochemical properties of millet starch through fermentation or chemical modification [9,10,11], such as using *Lactobacillus plantarum* to regulate its physicochemical and functional properties [9,10]; these efforts often focus on targeted traits without fully addressing the native starch characteristics and the inherent interdependence between starch structure and function in a native context. Some studies have demonstrated that such structure–physicochemical–function relationships are critical; for example, the swelling power of jackfruit starch is influenced by both chain interactions and particle size (*r* = 0.91, *p* < 0.01) [12], and amylose content in potato and rice starch has been shown to impact gelatinization by modulating the crystalline order [13,14]. Liang et al. [15] found that the digestibility of barley starch is closely linked to both amylose content and crystallinity. Therefore, it is necessary to understand the structural, physicochemical and functional properties to determine the distinct performance of FMSs.

Due to the varying amylose content, FMSs can be classified into waxy (with lower amylose content) and non-waxy types (with higher amylose content). In this study, five types of non-waxy FMSs were selected as experimental materials, with one waxy FMS serving as a control. The six non-waxy foxtail millet starches were derived from different varieties cultivated in the same location and stored in a vacuum desiccator at room temperature. This selection allowed us to focus on inherent varietal differences in structural, physicochemical, and functional properties, minimizing the influence of environmental factors. The crystalline structure and granule morphology were characterized using X-ray diffraction and scanning electron microscopy, while physicochemical and functional properties, including solubility, swelling power, gelatinization, thermal behavior, and in vitro digestibility, were systematically analyzed with further correlation analysis. This study revealed the particular properties of FMSs, examining their structural, physicochemical, and functional properties, aiming to provide a scientific basis for the effective utilization of foxtail millet starch and to explore its potential applications in functional foods and other starch-based materials, thereby contributing to improved utilization of underexploited cereal crops.

## 2. Materials and Methods

### 2.1. Materials

Five types of non-waxy foxtail millet (Heixiaomi, Lvxiaomi, Qinzhouhuang, Jingu21, Bailianggu) and one waxy foxtail millet (Hongjiugu) were obtained from the Agricultural Technology Promotion Center of Shenmu in Yulin, China. All foxtail millet varieties were grown at the experimental open farm of Agricultural Technology Promotion Center of Shenmu in Yulin, China (38°83′ N, 110°51′ E). Six FMSs were successfully extracted following the wet milling method reported by Ji et al. [16], named N-HXMS, N-LXMS, N-QZHS, N-JG21S, N-BLGS, and W-HJGS. The extracted starches had a purity of no less than 95% and were stored in a vacuum desiccator at room temperature. All the chemicals used were of analytical grade.

### 2.2. Proximate Composition

The total starch content was analyzed using a commercial total starch assay kit (Megazyme, International Ireland Ltd., Wicklow, Ireland). In brief, the starch in the sample was gelatinized by heating, partially hydrolyzed with thermostable α-amylase, and then completely hydrolyzed to glucose using amyloglucosidase. The released glucose was subsequently quantified spectrophotometrically, and the total starch content was calculated. The crude protein, lipid, and ash contents were measured following the procedures published by AOAC [17]. Briefly, the protein content was determined by the Kjeldahl method with a nitrogen-to-protein conversion factor of 6.25; the lipid content was measured by Soxhlet extraction using petroleum ether as the solvent; and the ash content was determined by incinerating the sample in a muffle furnace until a constant weight was obtained.

### 2.3. Amylose Content (AC)

The amylose content of the FMSs was measured according to an amylose–iodine complex-based colorimetric method following the AACC method 61–03.01 [18]. Then, 100 mg starch was mixed with 1 mL 95% ethanol and 9 mL 1 M NaOH, heated at 100 °C for 15 min, then cooled to room temperature and diluted to 100 mL with distilled water. In addition, 500 μL of the dispersion was mixed with 200 μL iodine solution and 100 μL 1 M acetic acid, and diluted to 10 mL with distilled water. After mixing and standing in the dark for 20 min, absorbance was measured at 620 nm.

### 2.4. Molecular Weight (M_w_)

Briefly, 20 mg starch (dry basis) was mixed with 1 mL 0.1 M NaNO_3_ solution and heated at 100 °C for 15 min to ensure complete dispersion. A high-performance liquid phase (HPLC, Model 410, Waters, Milford, MA, USA) coupled with a differential refractive detector and two analytical columns (Ultrahydrogel LInear 300 mm × 7.8 mm and Ultrahydrogel 120, 300 mm × 7.8 mm, Waters, Tokyo, Japan) was employed to determine the molecular weight of the samples, as reported [19].

### 2.5. Granule Structure

The granule structure of FMSs was evaluated based on their microstructure morphology and particle size [9]. Surface morphology was observed using scanning electron microscopy (Nova Nano SEM-450, FEI, Hillsboro, OR, USA), where starch samples were coated with a thin layer of gold to improve conductivity, and images were obtained under an accelerating voltage of 5 kV. Particle size distribution was determined using a laser diffraction particle size analyzer (ZEN3600, MALVERN, Malvern, UK). Prior to measurement, starch samples were dispersed in deionized water to ensure complete dispersion and provide a comprehensive assessment of particle size distribution.

### 2.6. Relative Crystallinity

According to the methods reported by You et al. [19], the crystalline structure of FMS was estimated using an X-ray diffractometer (D8 ADVANCE A25, Bruker, Karlsruhe, Germany) with a diffraction angle (2θ) from 5° to 45° with a step of 0.02°. The relative crystallinity of starches was calculated based on the amorphous baseline subtraction method reported by Zhang et al. [20], using Jade software (Version 6.5, Materials Data, Inc., Livermore, CA, USA).

### 2.7. Short-Range Molecular Order

Briefly, 3 mg starch samples were mixed with KBr at a ratio of 1:100 (*v*/*v*) and pressed into sheets based on Babu et al.’s methods [11]. Fourier transform infrared spectroscopy (Vetex70, Bruker, Germany) was carried out to determine the short-range molecular order of the samples. The spectra were obtained using a pure KBr sheet as background under scanning from 4000 to 400 cm^−1^ with a resolution of 4 cm^−1^.

### 2.8. Water Solubility (S_w_) and Swelling Power (S_p_)

Based on the methods reported by previous studies [7], *S_w_* and *S_p_* of the FMSs were measured with a slight modification. Briefly, 0.25 g FMS was dissolved in 25 mL distilled water and vortexed thoroughly to ensure uniform suspension. The suspension was heated at 90 °C with continuous shaking, cooled, and centrifuged (3500 rpm, 15 min) to collect the supernatant, while the precipitate was immediately weighed and recorded as *m*_0_. The collected supernatant was oven-dried at 105 °C to a constant weight and weighed, and the mass was recorded as m_1_. The *S*_w_ and *S*_p_ were assessed using the following equations:

$$S_{w}(\%)=\frac{m_{1}}{0.25}\times 100$$

$$S_{p}(g/g)=\frac{m_{0}}{0.25\times (100-S_{w})}$$

where *m*_0_ represents the precipitate weight (g), and *m*_1_ represents the dried supernatant weight (g).

### 2.9. Thermal Properties

Briefly, 3 mg FMS was accurately weighed and mixed with 9 μL distilled water in a sealed crucible to prevent moisture loss following the method of Soler et al. with some modifications [21]. The crucible was heated from 30 °C to 110 °C at a rate of 10 °C/min using differential scanning calorimetry (Waters, Q2000, Milford, MA, USA) to determine its gelatinization properties. During scanning, an empty sealed crucible was used as the reference. The scanned samples were kept at 4 °C for 7 days before being re-scanned by the same method to analyze the retrogradation properties.

### 2.10. Pasting Properties

FMS suspensions (14% *w*/*w*, 28 g) were heated using a rapid visco-analyzer (Perten, Tech Mastet, Stockholm, Sweden) to evaluate the pasting properties of the samples. The RVA program reported by Du et al. [22] was used. Briefly, an 8% starch suspension was equilibrated at 50 °C for 1 min, followed by heating to 95 °C at a rate of 6 °C/min. The temperature was then maintained at 95 °C for 5 min to ensure complete gelatinization, and subsequently cooled to 50 °C at the same rate. Throughout the analysis, the paddle rotated at a constant speed of 160 rpm, except for the initial 10 s, during which the speed was temporarily increased to 960 rpm to ensure uniform dispersion of starch granules. This program allowed for the assessment of key pasting parameters such as peak viscosity, breakdown, final viscosity, setback, and pasting temperature.

### 2.11. Rheological Properties

Based on the method reported by Zhao et al. with minor modifications [23], the rheological behavior of FMSs was assessed using a TA Instruments rheometer (DHR-1, Waters, Milford, MA, USA) equipped with a Peltier temperature control system and a 40 mm diameter parallel plate configuration. A 5% (*w*/*v*) gelatinized starch gel was carefully loaded onto the lower Peltier plate to ensure a fixed gap of 1000 μm.

#### 2.11.1. Steady Shear Test

To evaluate the steady shear flow behavior, a shear rate sweep was performed while the shear rate was gradually increased from 0.1 s^−1^ to 1000 s^−1^ and then decreased back to 0.1 s^−1^. Throughout the test, the apparent viscosity and shear stress were continuously recorded to capture the shear-thinning behavior and hysteresis between the upward and downward curves. The ascending flow curve was fitted using the Herschel–Bulkley model [7]:

$$\tau =\tau_{0}+{K\gamma }^{n}$$

where *τ* is the shear stress (Pa), *τ*_0_ is the yield stress (Pa), *K* is the consistency coefficient (Pa·sⁿ), *γ* is the shear rate (s^−1^), and *n* is the flow behavior index.

#### 2.11.2. Steady Recovery Test

The shear recovery behavior of FMS was evaluated at 25 °C using a three-step shear program [24], which included alternating low- and high-shear conditions. The samples were sheared at 1 s^−1^, 100 s^−1^, and 1 s^−1^ for 60 s, 120 s, and 180 s, respectively. The shear recovery capability was quantified by calculating the ratio of the average apparent viscosity during the first 30 s of the third phase to the average apparent viscosity in the first phase.

### 2.12. In Vitro Digestibility

Following a slightly modified version of the method by Du et al. [22], the in vitro digestibility of FMS was evaluated. In brief, 500 mg starch was dispersed in 10 mL 0.1 M sodium acetate buffer (pH 5.2), and gelatinized by heating in a boiling water bath for 30 min. After cooling to 37 °C for 10 min, 2.5 mL composite enzyme containing α-amylase (3000 U/mL) and amyloglucosidase (2500 U/mL) was added to initiate hydrolysis under shaking conditions at 37 °C. During digestion (shaking in a water bath at 37 °C), 4 mL ethanol was mixed with 0.5 mL enzymatic reaction at 20 and 120 min and centrifuged (3500 rpm, 5 min), and the glucose content of the supernatant was measured using the DNS method. Rapidly digestible starch (RDS), slowly digestible starch (SDS), and resistant starch (RS) contents were calculated according to following equations, respectively:

$$RDS\;(\%)=\frac{(G_{20}-FG)\times 0.9}{TS}\times 100$$

$$SDS\;(\%)=\frac{(G_{120}-G_{20})\times 0.9}{TS}\times 100$$

$$RS\;(\%)=\frac{TS-RDS-SDS}{TS}\times 100$$

### 2.13. Statistical Analysis

All experiments were repeated three times, and the results are expressed as means ± standard deviation. All data was analyzed using IBM SPSS Statistics 20 (IBM Corp., Armonk, NY, USA). One-way analysis of variance (ANOVA), followed by Duncan’s multiple range test, was applied to determine significant differences (*p* < 0.05). The data was normalized for Pearson’s correlation analysis, which was conducted using OriginPro 2023b (OriginLab Corp., Northampton, MA, USA).

## 3. Results and Discussion

### 3.1. Proximate Composition

The proximate compositions of FMS are presented in Table 1. The protein, lipid, and ash contents of all samples were relatively low, while the total starch content was generally high. The protein content varied between 0.33% and 0.81 wt%, with N-QZHS showing the highest level, which might partially influence its pasting and retrogradation behavior through protein–starch interactions [25]. The lipid content ranged from 0.22% to 0.61%, with N-BLGS and W-HJGS showing relatively higher lipid levels, which could affect thermal transitions [26]. The ash content (0.08–0.35%) showed significant differences among varieties, with N-LXMS exhibiting the lowest mineral residue and N-JG21S the highest. These minor variations could contribute to differences in physicochemical and functional properties observed among the FMSs.

### 3.2. Amylose Content (AC) and Molecular Weight (M_w_)

As shown in Table 1, compared with W-HJGS (0.88%), non-waxy foxtail millet starches (FMSs) exhibited a significantly higher AC content, which varied significantly across different varieties. N-BLGS exhibited the highest AC (34.04%), while N-QZHS had the lowest (32.40%). Similar findings were reported by Yang et al. [27], who found that the AC of non-waxy and waxy FMS were 36.70% and 2.50%, respectively. Differences in amylose levels were primarily attributed to genetic variation and environmental factors, while AC could affect the structural, functional and digestive properties of starch [28]. Based on the different AC of FMSs, it could be selectively utilized in various applications requiring specific properties. The non-waxy FMSs were proven to be a promising candidate for novel biomedical materials, while waxy FMS is highly suitable for use as a thickener and in frozen convenience foods [29].

The molecular weight (*M_w_*) and the full width at half maximum (FWHM) of all FMSs are shown in Table 1. *M_w_* of the waxy FMS (W-HJGS) was significantly higher that of the non-waxy FMSs (*p* < 0.05). No significant differences in *M_w_* were observed among the five non-waxy FMSs. The FWHM is considered to be a representative indicator of molecular weight distribution, with a larger FWHM reflecting a broader and more heterogeneous distribution [30]. Based on previous studies, a narrower molecular weight distribution might facilitate starch crystallization [31]. Among all FMS, N-BLGS displayed the narrowest molecular weight distribution. A significant positive association between molecular distribution uniformity and crystallinity (*r* = 0.930 **) was also observed in this study.

### 3.3. Granule Structure

The granule size distribution affects the physicochemical properties of the starches [32]. The particle size distribution of FMS ranged from 0.04 to 22.73 μm, with an average particle size between 8.39 and 8.95 μm (Table 2), which was consistent with the findings reported by Qi et al. [33]. Variations in particle size were observed among different non-waxy FMS, with N-BLGS exhibiting the largest mean diameter, while N-QZHS had the smallest. Except for N-QZHS, non-waxy varieties tended to have slightly larger average granule particle sizes. This phenomenon aligned with the results of previous studies, which indicated that a higher amylose content might be associated with larger starch granules, possibly due to differences in biosynthetic pathways [34]. The granule size of FMSs was comparable to that of foxtail millet starch (8.44–8.68 μm) [9], but substantially smaller than that of maize starch (15.45 μm) and potato starch (42.26 μm), as reported by a previous study [28].

**Figure 1:**
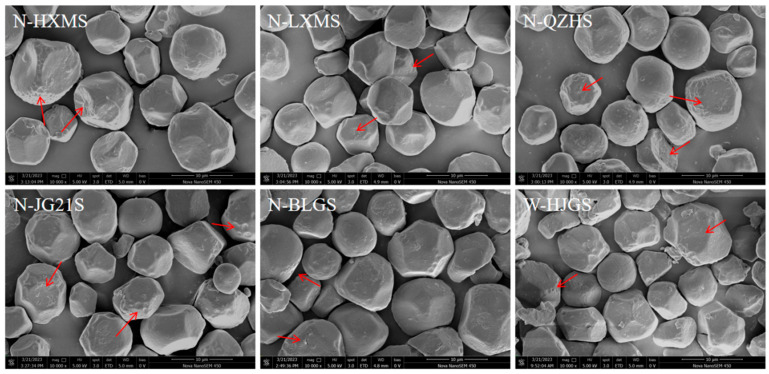
SEM images of different FMS. The red arrow points to FMS granule pores.

The granule morphology of waxy and non-waxy FMSs was similar, while the waxy FMS granules exhibited slightly more surface pores (Figure 1). Most FMS granules were polygonal in shape, with a few appearing spherical. A small number of damaged granules was observed, likely due to the mechanical effects during starch extraction processing. In addition, environmental factors, processing conditions, and the location of starch granules within the endosperm could also influence granule morphology [35]. Some granules’ surfaces exhibited concave shapes, possibly due to compression from protein bodies [36]. SEM images of FMSs revealed randomly distributed pores on the granule surfaces, which might serve as accessible sites for enzymes and other reagents to diffuse into the granule, a feature similar to that observed in maize starch but absent in potato starch, which is potentially characteristic of A-type cereal starch [37]. Such surface properties might enhance enzyme accessibility, facilitating starch digestion through an ‘inside-out’ digestion model [36,38]. Moreover, the presence of pores and channels could enable water and oxhydryl to penetrate into the granules, therefore disrupting the amorphous regions and reducing the steric restriction of amylose chains, contributing to improved hydration and swelling properties [36]. This is beneficial in applications requiring high water-binding capacity, such as food gels and thickeners [36].

### 3.4. X-Ray Diffraction (XRD)

**Figure 2:**
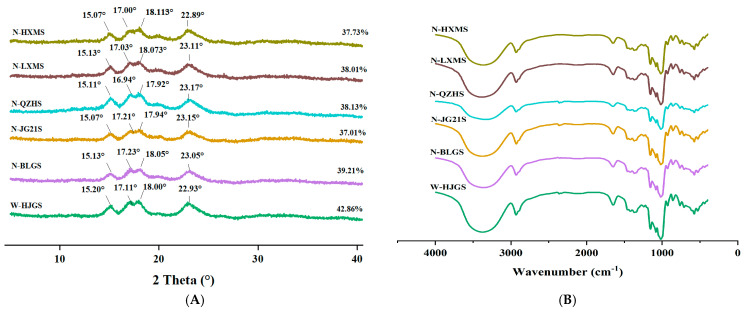
XRD patterns (**A**) and FTIR spectrum (**B**) of different FMSs.

All FMSs exhibited characteristic diffraction peaks at 15°, 17°, 18°, and 23°, corresponding to an orthorhombic crystalline structure with space group *B*2 (or *I*222), referred to as “A-type” starch (Figure 2A) [39]. This observation was consistent with the findings of previous research [23,36]. The relative crystallinity (RC) of the FMSs ranged from 37.01% to 42.86% (Figure 2A), which was comparable to that reported for corn starch (36.05%) and markedly higher than that of potato starch (25.68%) [7]. Such differences in RC among starches could generally be attributed to variations in amylose content, molecular organization, and crystalline type, which, in turn, affect their functional properties, such as gelatinization, pasting behavior, and enzymatic digestibility. Notably, the RC of non-waxy FMSs was significantly lower than that of the waxy FMS (*p* < 0.05). Among the non-waxy FMSs, N-BLGS exhibited the highest RC (39.21%), whereas N-JG21S had the lowest RC (37.01%). In general, amylose is considered to reside primarily in the amorphous regions of starch granules, and a higher amylose content is typically associated with lower relative crystallinity [28]. However, N-BLGS exhibited both high amylose content and relatively high crystallinity. This might be attributed to the fact that starch crystallinity was governed by multiple structural factors, including amylopectin chain length distribution, crystal size, double-helix packing, and their intermolecular interactions [40]. These factors may act synergistically, thereby resulting in the comparatively higher crystallinity observed in N-BLGS [40]. In addition, these variations in amylose content and different distribution of amylopectin chains might modulate the packing efficiency of double helices in the orthorhombic lattice [39].

### 3.5. Short-Range Molecular Order

As illustrated in Figure 2B, the FTIR spectra of the six FMSs showed similar profiles, suggesting that the major functional groups present in different FMSs were consistent [7]. According to Romero-García et al. [41], the broad absorption band near 3306 cm^−1^ is typically attributed to O–H stretching vibrations. Bands near 2929 cm^−1^ and 2883 cm^−1^ are generally assigned to the asymmetric and symmetric stretching modes of –CH and –CH_2_ moieties, respectively. A distinct band around 1329 cm^−1^ is related to the in-plane bending vibration of CH_2_ groups. The absorption band at 1147 cm^−1^ corresponds to stretching vibrations involving C–C and C–O–C bonds, while the bending vibrations of the COH groups are usually associated with the band at 1079 cm^−1^. Around 1015 cm^−1^, a combination of C–C and C–O stretching, along with COH in-plane bending vibrations, can be observed. Bands near 994 cm^−1^ and 926 cm^−1^ are typically linked to the bending vibrations of C-O-C bonds in starch backbone, CCH, and COH groups. The absorption bands at 1047, 1022, and 1000 cm^−1^ fall within the 1100–900 cm^−1^ region, which is known to be sensitive to changes in starch structure [42]. Previous studies have associated the 1047 cm^−1^ band with more ordered regions (e.g., double helices), and the 1022 cm^−1^ band with less ordered, amorphous regions [28,42,43,44]. The absorbance ratio *R*_1047/1022_ is thus often used as a qualitative index of short-range order [42,43,44]. As shown in Table 2, there were differences in the degree of short-range order of different types of FMSs (*p* < 0.05). Among five types of non-waxy FMSs, N-BLGS showed the highest *R*_1047/1022_ value (0.853), while N-QZHS exhibited the lowest (0.767). The *R*_1047/1022_ ratio (0.863) of W-HJGS was significantly higher than that of the non-waxy starches (*p* < 0.05). Similar observations had been found in waxy and normal wheat starches reported by Sun et al. [32], who found that starches with lower AC and higher crystallinity were more likely to form a more ordered short-range structure. Additionally, researchers also demonstrated that more amylopectin long branches could promote the creation of a more ordered short-range structure [35]. The ordered short-range structure of starch was influenced by its botanical source, amylose content, long amylopectin branch content, and crystallinity [7,28,32].

It should be emphasized that the quantification of starch crystallinity based on XRD and FTIR is highly method-dependent. Consequently, these results are more appropriate for comparative evaluation rather than absolute quantification. Therefore, the crystallinity data presented in this study should be interpreted with caution and considered in combination with the following structural and functional analyses.

### 3.6. Water Solubility (S_w_) and Swelling Power (S_p_)

The water solubility (*S_w_*) and swelling power (*S_p_*) of starches are shown in Table 3, reflecting the extent of interaction between starch and water [45]. W-HJGS exhibited significantly higher *S_w_* (92.30%) and *S_p_* (22.76 g/g) than those of non-waxy FMSs (8.57–10.01%, 18.79 g/g–20.22 g/g) (*p* < 0.05). Among the non-waxy starches, N-QZHS had the lowest *S*_w_ (8.57%), whereas N-HXMS showed the highest *S*_p_ (20.2 g/g), indicating structural differences even within the same starch type. As AC was the key parameter affecting starch *S_w_*, the higher water solubility of W-HJGS was mainly due to its lower AC [46]. Specifically, in W-HJGS, which contained little amylose, the enhanced solubility can be attributed to the looser molecular packing and the greater exposure of hydrophilic groups, facilitating stronger interactions with water. Additionally, *S_p_* (*r* = −0.921 **) and *S_w_* (*r* = −0.998 **) showed significant correlations with AC, which might be related to the fact that the higher amylose content restricts starch granule hydration and expansion by reinforcing the internal molecular network due to its compact and hydrogen-bond-rich molecular architecture [7]. The variations in *S*_w_ and *S*_p_ among non-waxy FMSs may also be influenced by other structural factors, such as granule size, crystalline structure, and amylopectin chain length [7].

### 3.7. Thermal Properties

**Figure 3:**
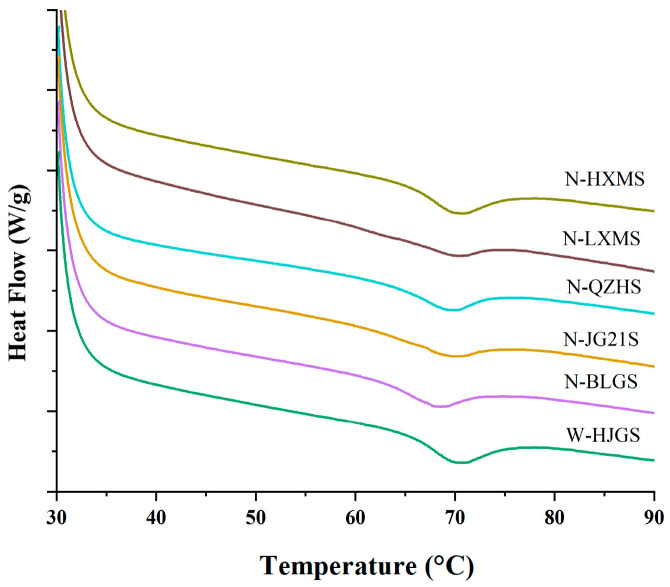
DSC curves of different FMSs.

As depicted in Table 4, significant differences were observed in the onset (T_o_), peak (T_p_), conclusion (T_c_) temperatures, gelatinization enthalpy (ΔH), and retrogradation ratio (*R_r_*) of different FMSs (*p* < 0.05). Compared with W-HJGS (Figure 3), non-waxy FMS exhibited lower T_o_ (62.85–64.32 °C), T_p_ (68.02–69.76 °C), T_c_ (75.95–78.40 °C), and ΔH (7.19–11.05 J/g). These phenomena could be attributed to the higher AC, lower RC and lower *R*_1047/1022_ value in non-waxy samples. Similar findings were reported by Chang et al.; the waxy proso millet starch showed elevated crystallinity and a more ordered double-helix structure, which required a higher thermal input for gelatinization [28]. Specifically, the higher-ordered crystalline regions restricted water penetration and gelatinization, while the tight amylose chains required more energy to break their interactions and initiate gel formation [47,48]. Compared with non-waxy proso millet starch (T_o_ = 66.19 °C, T_p_ = 69.62 °C, T_c_ = 76.68 °C), which was reported by Chang et al. [28], the non-waxy FMSs exhibited lower T_o_, while T_p_ and T_c_ were similar, indicating that non-waxy FMSs were more easily gelatinized than non-waxy proso millet starch. A similar trend was observed for waxy FMS and waxy proso millet starch, and this similar trend could be attributed to varietal differences. In addition, the T_o_ and T_p_ of FMS were generally intermediate between those of corn starch and potato starch reported by our previous study [28], while Tc was higher than that of potato starch but comparable to that of corn starch. These results could serve as a reference for potential modification and application as a partial substitute for corn starch in food systems. The retrogradation ratio (*R_r_*) of non-waxy FMSs was significantly higher than that of waxy FMS (*p* < 0.05). Among the non-waxy FMSs, the highest retrogradation rates of N-JG21S (34.40%) and N-BLGS (34.32%) were likely due to their relative higher AC. The correlation analysis also revealed a similar trend, showing a significant positive correlation between AC and the retrogradation rate (*r* = 0.946 **), which was consistent with previous findings that higher amylose levels tend to promote retrogradation in cooked rice [49]. This might be related the liner structure of amylose, which more easily formed hydrogen bonds with others. And the highly branched structure of amylopectin could retard hydrogen bond formation. In addition, the retrogradation process could influence starch crystallinity, as the recrystallization of amylose and amylopectin chains altered the crystalline structure during storage [50]. Therefore, when considering retrograded FMSs for product applications, the impact of changes in crystallinity should be carefully taken into account as it could affect the texture, stability, and digestibility of the final product.

### 3.8. Pasting Properties

The pasting properties of FMSs are presented in Table 5, showing significant differences (*p* < 0.05). Compared with all non-waxy FMSs, W-HJGS exhibited a significantly lower pasting temperature (PT), final viscosity (FV) and setback (SB), and higher breakdown (BD) (*p* < 0.05), indicating that the waxy starch tended to swell and gelatinize at a lower temperature with minimal retrogradation. In contrast, the higher PT, FV and SB of non-waxy starches reflect their enhanced thermal, shear resistance, and retrogradation tendency, likely resulting from their elevated amylose levels [28]. W-HJGS exhibited the lowest PT, indicating its ability to interact with water and swell to a certain viscosity at relatively lower temperatures. This behavior was consistent with its higher solubility and swelling power (Table 3). Among the non-waxy varieties, N-BLGS showed the highest PT, suggesting that it was difficult to adsorb water and swell, thereby making gelatinization more difficult, while N-LXMS had the lowest. Higher AC levels tended to suppress starch swelling and gelatinization, often resulting in increased PT. N-HXMS and N-LXMS exhibited higher PV compared with W-HJGS (*p* < 0.05), which aligned with the findings of Zhong et al. [14]. According to their study, higher crystallinity and more extensive double-helix content might reduce PV [14]. In contrast, starches with thicker crystalline lamellae and/or thinner amorphous layers tended to exhibit higher peak viscosity. In our study, RC exhibited a negatively correlation with FV (*r* = 0.986 **) and SB (*r* = 0.991 **), implying that a more crystalline starch structure may enhance gel stability, which was consistent with a previous study [51]. This was because higher crystallinity restricted granule swelling and rupture during pasting, leading to a more uniform and stable gel network, which reduced retrogradation tendency [51]. Notably, the underlying mechanisms are likely multifactorial, as starch viscosity is influenced by botanical origin, processing conditions, and the fine structure of amylopectin, including chain-length distribution [6]. In addition, compared with other common starches [28], the PT of FMSs was higher than that of potato starch (69.52 °C) but lower than that of corn starch (77.42 °C), the PV was lower than that of potato starch (6443 cP) but similar to that of corn starch (2822 cP), and the BD was lower than that of potato starch (4377 cP) but higher than that of corn starch (2822 cP), indicating intermediate swelling and thermal stability. Non-waxy FMSs showed FV higher than that of both potato (2316 cP) and corn starch (3051 cP), while waxy FMS exhibited lower FV than that of these starches. In terms of retrogradation tendency, the SB of non-waxy FMSs was higher than that of both corn (1162 cP) and potato starch (249 cP), whereas waxy FMSs exhibited similar SB value with potato starch. These findings suggest that FMSs could be directionally modified in the future for specific food applications.

### 3.9. Rheological Properties

**Figure 4:**
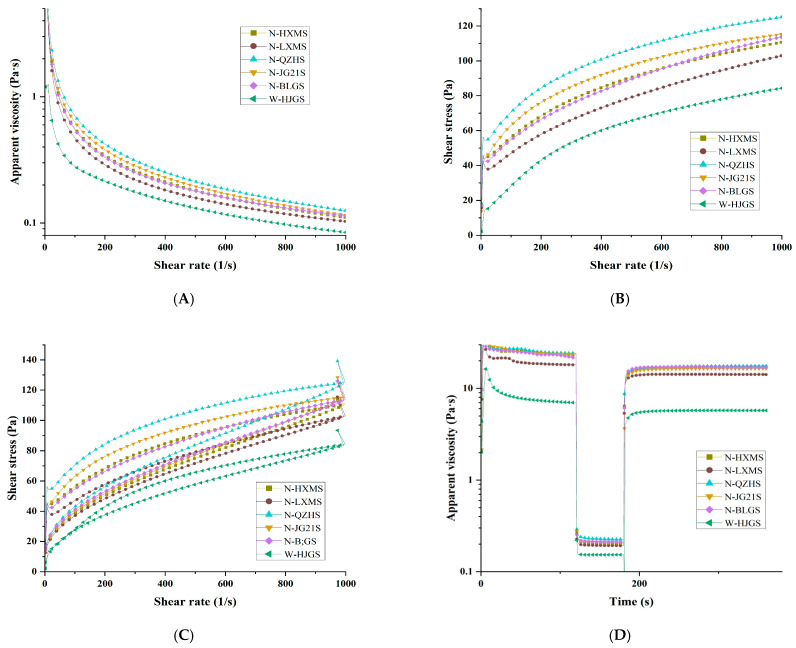
Apparent viscosity plot against shear rate (**A**), shear stress against shear rate (**B**,**C**), recovery curve (**D**) of different FMSs.

The rheological properties of FMS are shown in Figure 4, while the upward curves of shear stress versus shear rate for all FMSs (Figure 4B) fitted well to the Herschel–Bulkley model, with an *R^2^* from 0.975 to 0.996 (Table 6), indicating excellent model applicability. Figure 4A shows that all FMS gels exhibited shear-thinning behavior, a characteristic of pseudoplastic non-Newtonian fluids, with apparent viscosity decreasing as the shear rate increased [7], while Table 6 further demonstrated their pseudoplastic characteristics (*n* < 1). Among the six FMSs, N-QZHS consistently showed the highest viscosity across the entire shear rate range, followed by N-JG21S, N-HXMS, N-BLGS, N-LXMS, and W-HJGS (Figure 4A). W-HJGS, with the lowest AC of 0.88%, had the lowest viscosity despite having the highest crystallinity. This phenomenon was due to the lack of amylose, which limited the formation of an extended, entangled molecular network for resisting starch molecular flowing [52]. In contrast, the non-waxy FMSs contained relatively high AC, enhancing intermolecular interactions and entanglement, thus increasing viscosity [7,28,52]. The yield stress (τ_0_) represented the minimum shear stress required to initiate flow [52]. For W-HJGS, the lowest τ_0_ suggested it could flow readily without the need for an initial yield stress (Table 6), likely due to its extremely low AC and weak internal structure. In contrast, all non-waxy starches showed positive τ_0_ values, indicating their stronger structural resistance compared with the waxy FMS [53]. The consistency index (*K*) reflected the resistance to shear and flow. The relative lower *K* value and higher n value of N-HXMS, N-LXMS, and N-BLGS informed their poorer pseudoplasticity.

The thixotropic curves are depicted in Figure 4C, thixotropic curves represent the structural stability of starch gels under shear, with the size of the hysteresis loop indicating the energy required to disrupt the starch’s internal network [7]. The mean thixotropic loop areas of the FMSs followed the same order as their apparent viscosity in Figure 4A (N-QZHS > N-JG21S > N-HXMS > N-BLGS > N-LXMS > W-HJGS), suggesting a close relationship between viscosity and structural stability. The lowest thixotropic curves were observed in the waxy FMS (W-HJGS), showing its poorest structural stability. The shear structure recovery was generally evaluated by the ratio of apparent viscosity in the first 30 s of the third processing to that in the first processing (Figure 4D). As shown in Table 6, W-HJGS had the lowest recovery rate (48.65%), indicating the weakest structural restoration after shear, while N-BLGS exhibited the highest recovery rate (58.62%), reflecting the strongest ability to rebuild its internal network [24].

Interestingly, granule size showed a significantly negative correlation with shear recovery (*r* = −0.898 *). Further investigations could focus on the mechanistic understanding of whether granule size could influence the breakdown and rebuilding of microstructures during and after shear. In addition, several researchers reported that the rheological parameters could be affected by AC, internal chain structure of amylopectin, and the content of long amylopectin branches [7,28,53,54].

### 3.10. In Vitro Starch Digestibility

The RDS, SDS, and RS contents of different FMSs are shown in Table 7. All non-waxy FMSs exhibited significantly lower contents of RDS compared with the waxy FMS, which was closely associated with their higher amylose content (Table 1). According to the study of Govindaraju et al. [40], enzymatic digestibility of starch was significantly negatively correlated with AC, which played a critical role in determining starch hydrolysis behavior, as α-amylase primarily attacked α-1, 4 glycosidic bonds in amorphous regions of starch granules. Non-waxy FMSs, with higher AC, formed dense structures that hindered enzyme access to α-1,4 glycosidic bonds, resulting in lower RDS contents (38.64–49.74%) and higher SDS (28.03–33.66%) and RS contents (16.61–31.17%). Notably, starches with higher amylose content generally exhibited higher resistant starch (RS) levels, which was consistent with previous studies [35,55]. In particular, higher amylose content could lead to reduced granule swelling, thereby limiting enzyme penetration and ultimately promoting the formation of resistant starch (RS) [55]. Pan et al. reported that even when the ACs in six rice starch varieties were similar, their digestibility varied significantly due to differences in molecular and crystalline structures [56]. The digestibility characteristics of non-waxy FMSs also differed significantly among varieties (*p* < 0.05). N-JG21S and N-BLGS exhibited higher RDS levels, which might result in a rapid postprandial blood glucose increase. In contrast, N-LXMS showed lower RDS and higher RS contents, indicating greater resistance to enzymatic hydrolysis and making it suitable for foods designed for individuals with diabetes or obesity. In this study, the digestibility of starch was negatively correlated with AC(*r* = −0.851 *), while digestibility was also influenced by amylose-lipid complexes and molecular structure of amylopectin [28].

## 4. Conclusions

In this study, the structural, physicochemical, and functional properties of starches isolated from six Chinese foxtail millet varieties were investigated. All FMS granules were predominantly polygonal with few surface pores, showing an orthorhombic crystalline structure with space group B2 (or alternatively I222), referred to as “A-type” starch, and average particle sizes ranging from 8.39 to 8.95 μm. Significant differences were observed across all measured parameters, indicating their potential for diverse applications. For instance, N-LXMS, with its relatively high amylose and resistant starch contents, may serve as a preferred ingredient for functional foods targeting glycemic control. W-HJGS, exhibiting higher swelling power and solubility, is suitable as a thickener or binder. N-JG21S and N-LXMS have superior shear recovery, suggesting better structural resilience under mechanical processing, which could benefit texturizing and stabilizing applications such as 3D printing. These differences are closely associated with the intrinsic structural and physicochemical characteristics of starches while also being influenced by their botanical origin. In addition, the gelatinization and pasting properties of FMS are generally intermediate between those of corn starch (CS) and potato starch (PS), suggesting that targeted modification could enable FMSs to serve as an alternative to commonly used starches in various applications. In future research, we plan to investigate the impact mechanism of genotype–environment interactions on the stability of these starch traits. Building on these insights, we will further explore targeted modification strategies to optimize their functional properties and broaden their potential applications.

## Figures and Tables

**Table 1:** The proximate composition, amylose content, M_w_, and FWHM of FMSs.

Starch Sample	Moisture (%)	Total Starch (%)	Protein (%)	Lipid (%)	Ash (%)	Amylose Content (%)	*M_w_* (×10^5^ g/mol)	FWHM
N-HXMS	2.85 ± 0.11 ^d^	95.77 ± 0.24 ^ab^	0.52 ± 0.04 ^bc^	0.49 ± 0.02 ^a^	0.33 ± 0.04 ^a^	33.60 ± 0.12 ^a^	2.45 ± 0.09 ^b^	3.704 ± 0.010 ^b^
N-LXMS	3.60 ± 0.07 ^c^	95.56 ± 0.30 ^b^	0.46 ± 0.18 ^cd^	0.28 ± 0.09 ^b^	0.08 ± 0.01 ^c^	32.91 ± 0.04 ^b^	2.68 ± 0.05 ^b^	3.902 ± 0.010 ^a^
N-QZHS	2.51 ± 0.02 ^e^	96.28 ± 0.19 ^a^	0.81 ± 0.09 ^a^	0.22 ± 0.01 ^b^	0.17 ± 0.03 ^bc^	32.40 ± 0.15 ^b^	2.81 ± 0.09 ^b^	3.640 ± 0.005 ^c^
N-JG21S	3.93 ± 0.18 ^b^	95.05 ± 0.02 ^b^	0.33 ± 0.07 ^d^	0.32 ± 0.03 ^b^	0.35 ± 0.09 ^a^	33.71 ± 0.49 ^a^	2.08 ± 0.06 ^b^	3.704 ± 0.005 ^b^
N-BLGS	3.49 ± 0.05 ^c^	94.99 ± 0.17 ^b^	0.68 ± 0.10 ^ab^	0.61 ± 0.17 ^a^	0.18 ± 0.01 ^bc^	34.04 ± 0.04 ^a^	1.01 ± 0.02 ^b^	3.654 ± 0.005 ^c^
W-HJGS	4.44 ± 0.09 ^a^	94.38 ± 0.99 ^c^	0.37 ± 0.02 ^cd^	0.56 ± 0.11 ^a^	0.24 ± 0.10 ^b^	0.88 ± 0.00 ^c^	34.93 ± 3.71 ^a^	3.697 ± 0.005 ^b^

Results are mean ± standard deviation of triplicate analysis. Values followed by different letters in the same column are significantly different (*p* < 0.05). *M_w_*, molecular weight; FWHM, the full width at half maximum; R_1047/1022_, the ratio of intensity of two peaks at 1022 cm^−1^, and 1047 cm^−1^, respectively.

**Table 2:** The particle size distribution and *R*_1047/1022_ of different FMSs.

Starch Sample	Particle Size Distribution		*R*_1047/1022_
	Particle Size Distribution Range (μm)	Average Particle Size (μm)	
N-HXMS	0.16–18.86	8.94 ± 0.06 ^a^	0.843 ± 0.003 ^c^
N-LXMS	0.13–22.73	8.90 ± 0.12 ^ab^	0.785 ± 0.001 ^e^
N-QZHS	0.04–22.73	8.39 ± 0.65 ^b^	0.767 ± 0.008 ^f^
N-JG21S	0.13–22.73	8.68 ± 0.07 ^ab^	0.805 ± 0.000 ^d^
N-BLGS	0.04–18.86	8.95 ± 0.55 ^a^	0.853 ± 0.006 ^b^
W-HJGS	0.31–22.73	8.44 ± 0.14 ^ab^	0.863 ± 0.003 ^a^

The results are mean ± standard deviation of triplicate analysis. Values followed by different letters in the same column are significantly different (*p* < 0.05).

**Table 3:** The water solubility and swelling power of FMS.

Starch Sample	Water Solubility (%)	Swelling Power (g/g)
N-HXMS	10.01 ± 0.11 ^b^	20.22 ± 0.57 ^b^
N-LXMS	8.73 ± 0.43 ^cd^	19.87 ± 0.14 ^bc^
N-QZHS	8.57 ± 0.14 ^d^	18.86 ± 0.02 ^c^
N-JG21S	8.96 ± 0.23 ^cd^	18.79 ± 0.63 ^c^
N-BLGS	9.30 ± 0.21 ^c^	19.43 ± 0.58 ^bc^
W-HJGS	92.30 ± 0.09 ^a^	22.76 ± 0.28 ^a^

The results are mean ± standard deviation of triplicate analysis. Values followed by different letters in the same column are significantly different (*p* < 0.05).

**Table 4:** The thermal properties of different FMSs.

Starch Sample	T_o_ (°C)	T_p_ (°C)	T_c_ (°C)	ΔH (J/g)	*R_r_* (%)
N-HXMS	63.57 ± 0.00 ^bc^	69.57 ± 0.36 ^b^	78.40 ± 0.77 ^a^	9.73 ± 0.39 ^b^	32.48 ± 0.66 ^b^
N-LXMS	63.55 ± 0.69 ^bc^	69.76 ± 0.14 ^ab^	75.95 ± 1.06 ^c^	7.19 ± 0.87 ^c^	30.64 ± 0.10 ^c^
N-QZHS	64.32 ± 0.31 ^b^	69.54 ± 0.36 ^b^	77.85 ± 0.28 ^ab^	11.05 ± 0.42 ^b^	31.33 ± 0.02 ^bc^
N-JG21S	62.85 ± 0.29 ^c^	69.43 ± 0.13 ^b^	77.85 ± 0.05 ^ab^	10.39 ± 0.22 ^b^	34.40 ± 0.86 ^a^
N-BLGS	62.96 ± 0.33 ^c^	68.02 ± 0.04 ^c^	76.57 ± 0.64 ^bc^	10.27 ± 0.67 ^b^	34.42 ± 0.42 ^a^
W-HJGS	65.39 ± 0.30 ^a^	70.14 ± 0.01 ^a^	79.22 ± 0.53 ^a^	12.58 ± 0.07 ^a^	22.78 ± 1.13 ^d^

The results are mean ± standard deviation of triplicate analysis. Values followed by different letters in the same column are significantly different (*p* < 0.05).

**Table 5:** The pasting properties of different FMS.

Starch Sample	Pasting Temperature (°C)	Peak Viscosity (cP)	Breakdown (cP)	Final Viscosity (cP)	Setback (cP)
N-HXMS	82.40 ± 0.05 ^a^	3198 ± 5 ^a^	1303 ± 25 ^b^	3676 ± 46 ^a^	1782 ± 26 ^a^
N-LXMS	78.37 ± 0.03 ^d^	3161 ± 23 ^a^	1251 ± 89 ^bc^	3639 ± 49 ^a^	1729 ± 38 ^b^
N-QZHS	79.13 ± 0.04 ^c^	2839 ± 52 ^c^	1163 ± 12 ^cd^	3291 ± 87 ^d^	1614 ± 23 ^c^
N-JG21S	79.98 ± 0.04 ^b^	2847 ± 25 ^bc^	1042 ± 1 ^e^	3390 ± 21 ^c^	1585 ± 5 ^c^
N-BLGS	79.10 ± 0.00 ^c^	2932 ± 61 ^b^	1078 ± 46 ^de^	3491 ± 29 ^b^	1636 ± 14 ^c^
W-HJGS	76.70 ± 0.00 ^e^	2874 ± 54 ^bc^	1638 ± 54 ^a^	1495 ± 14 ^e^	259 ± 15 ^d^

Results are mean ± standard deviation of triplicate analysis. Values followed by different letters in the same column are significantly different (*p* < 0.05).

**Table 6:** The rheological properties of different FMS.

Starch Sample	*τ*_0_ (Pa)	*K* (Pa·s ^n^)	*n*	*R* ^2^	Hysteresis Loop Relative Area (kPa/s)	Shear Recovery (%)
N-HXMS	22.32 ± 1.48 ^b^	5.40 ± 0.12 ^c^	0.41 ± 0.01 ^d^	0.993	12.43 ± 0.79 ^c^	53.29 ± 3.43 ^b^
N-LXMS	26.59 ± 0.67 ^a^	1.59 ± 0.28 ^f^	0.56 ± 0.00 ^a^	0.995	4.92 ± 0.33 ^e^	57.97 ± 0.61 ^a^
N-QZHS	15.17 ± 0.91 ^c^	14.89 ± 0.36 ^b^	0.29 ± 0.00 ^e^	0.992	16.77 ± 1.28 ^a^	51.73 ± 2.39 ^c^
N-JG21S	5.00 ± 0.01 ^d^	16.61 ± 1.03 ^a^	0.28 ± 0.00 ^e^	0.995	14.84 ± 2.11 ^b^	49.15 ± 0.86 ^d^
N-BLGS	27.13 ± 1.18 ^a^	2.91 ± 0.41 ^d^	0.49 ± 0.01 ^c^	0.996	7.06 ± 0.52 ^d^	58.70 ± 1.88 ^a^
W-HJGS	0.00 ± 0.00 ^e^	2.28 ± 0.62 ^e^	0.53 ± 0.01 ^b^	0.975	3.67 ± 0.13 ^f^	48.22 ± 0.74 ^d^

The results are mean ± standard deviation of triplicate analysis. Values followed by different letters in the same column are significantly different (*p* < 0.05).

**Table 7:** The digestibility of different FMSs.

Starch Sample	RDS (%)	SDS (%)	RS (%)
N-HXMS	46.37 ± 0.18 ^c^	28.03 ± 0.14 ^c^	25.61 ± 0.04 ^c^
N-LXMS	38.64 ± 0.83 ^e^	30.20 ± 1.00 ^b^	31.17 ± 0.18 ^a^
N-QZHS	49.74 ± 0.46 ^b^	33.66 ± 0.33 ^a^	16.61 ± 0.13 ^e^
N-JG21S	42.21 ± 0.49 ^d^	33.19 ± 0.00 ^a^	24.61 ± 0.49 ^d^
N-BLGS	43.06 ± 0.24 ^d^	30.14 ± 0.25 ^b^	26.81 ± 0.00 ^b^
W-HJGS	58.58 ± 0.35 ^a^	27.41 ± 0.79 ^c^	14.01 ± 0.44 ^f^

The results are mean ± standard deviation of triplicate analysis. Values followed by different letters in the same column are significantly different (*p* < 0.05).

## Data Availability

The original contributions presented in this study are included in the article/Supplementary Materials. Further inquiries can be directed to the corresponding author.

## Supplementary Materials

The following supporting information can be downloaded at: https://www.mdpi.com/article/10.3390/foods14173034/s1, Table S1: Key properties of FMS and other starches, Table S2: The correlation of structural parameters and functional properties of FMS.
