# Supplementary material for: Structural, Physicochemical, and Functional Properties of Waxy and Non-Waxy Foxtail Millet Starches
Source: Foods. 2025 Aug 29;14(17):3034. doi: 10.3390/foods14173034 (PMC12427984; doi:10.3390/foods14173034)
Supplement: Supplementary file 1 [file foods-14-03034-s001.zip › foods-3839839-supplementary.pdf]

Table S1 Key properties of FMS and other starches

Starch source	Crystallinity type	Relative crystallinity (%)	Gelatinization temperature			Pasting properties					RS content (%)	Key reference
			To(°C)	Tp	Tc	PT	PV	FV	BD	SB		
non-waxy FMS	A-type	37.01-39.21	62.85-64.32	68.02-69.76	76.57-78.4	78.37-82.4	2839-3198	3291-3491	1042-1303	1585-1782	16.61-31.17	/
waxy FMS	A-type	42.86	65.39	70.14	79.22	76.7	2874	1495	1638	259	14.01	/
Potato starch	B-type	26.04	59.52	63	72.28	69.52	6443	2316	4377	249	9	[28]
Corn starch	A-type	35.81	67.57	70.08	76.72	77.42	2822	3051	932	1162	10.84	[28]
non-waxy proso millet starch	A-type	37.24	66.19	69.62	76.68	78.54	2956	3028	1404	1477	11.52	[28]
waxy proso millet starch	A-type	40.61	68.7	72.2	78.76	77.46	3506	2237	1698	429	9.38	[28]

Table S2 The correlation of structural parameters and functional properties of FMS.

		**AC**	***M_w***	**Average particle size**	**Relative crystalline**
Hydration properties	S_w	-0.998**	0.998**	-0.525	0.941**
	S_p	-0.921**	0.925**	-0.244	0.893*
	T_o	-0.854*	0.852*	-0.719	0.792
	R_r	0.946**	-0.947**	0.566	-0.872*
Thermal properties	BD	-0.895*	0.901*	-0.362	0.827*
	FV	0.986**	-0.983**	0.654	-0.923**
	SB	0.991**	-0.989**	0.596	-0.932**
	Shear recovery	-0.632	0.623	-0.898*	0.539
Digesibility	RDS	-0.851*	0.849*	-0.728	0.804

*, ** Correlation is significant at the 0.05, 0.01 level, respectively
